# Immediate Effects of a Jewett Brace on Posture and Dynamic Balance in Parkinson’s Disease-Associated Camptocormia: A Case Report

**DOI:** 10.7759/cureus.102612

**Published:** 2026-01-30

**Authors:** Chisato Nakamoto, Kyota Bando, Yohei Mukai, Yuji Takahashi, Kazuhiko Seki, Takatoshi Hara

**Affiliations:** 1 Department of Rehabilitation, National Center Hospital, National Center of Neurology and Psychiatry, Tokyo, JPN; 2 Department of Neurology, National Center Hospital, National Center of Neurology and Psychiatry, Tokyo, JPN; 3 Department of Neurophysiology, National Institute of Neuroscience, National Center of Neurology and Psychiatry, Tokyo, JPN

**Keywords:** camptocormia, case report, parkinson’s disease, postural control, trunk orthosis

## Abstract

Camptocormia (CC) in Parkinson’s disease (PD) is a disabling axial postural deformity characterized by involuntary thoracolumbar flexion. Although trunk orthoses are used for management, correcting the flexed posture may disrupt compensatory strategies for postural instability and potentially worsen dynamic balance. We report the case of a man in his 80s with PD (Hoehn & Yahr stage 3) who exhibited upper CC. We evaluated the immediate effects of a Jewett brace on posture and balance using a three-dimensional motion analysis system (Vicon, Oxford Metrics Ltd., Oxford, UK). Outcome measures included the trunk flexion angle during a three-minute standing task, Center of Gravity (COG) sway, and stepping responses during the Push and Release Test (PRT). Assessments were performed with the orthosis, followed by without it. The orthosis attenuated the increase in trunk flexion during standing (+5.1° with the orthosis vs. +12.3° without). No deterioration was observed in the COG sway range or PRT time variables (step initiation, first contact, and final stabilization) between conditions. In this case, the Jewett brace stabilized static posture without compromising dynamic balance or compensatory stepping responses. These findings suggest that trunk orthoses may be a safe intervention for postural correction in CC, provided that individual balance function is carefully assessed.

## Introduction

Camptocormia (CC) is a distinct axial postural abnormality characterized by involuntary thoracolumbar flexion that occurs during standing or walking and resolves in the supine position [1]. It is a common manifestation in Parkinson’s disease (PD), affecting approximately 3-17% of patients, and it significantly impairs activities of daily living (ADL) and quality of life (QOL) [2,3]. The Movement Disorder Society (MDS) consensus criteria define CC according to the fulcrum of flexion, distinguishing between upper and lower subtypes [1].

The etiology of CC is multifactorial, involving myopathic changes in the paraspinal muscles and central dysregulation of postural tone [4]. Treatment options include pharmacotherapy, botulinum toxin injections, deep brain stimulation, and rehabilitation; however, establishing high-quality evidence remains challenging [5].

Trunk orthoses are a non-pharmacological option intended to enable mechanical alignment of the spine [6]. However, their use raises an important clinical dilemma regarding whether CC reflects a compensatory strategy for postural instability. Recent computational studies suggest that a flexed posture in PD might serve to minimize postural sway under conditions of increased muscle tone [7]. If this hypothesis holds true, mechanically correcting the posture with an orthosis could disrupt this compensation, paradoxically increasing instability and the risk of falls. Conversely, other clinical studies indicate that CC itself contributes to gait instability and falls by lowering the center of mass excessively or limiting hip extension [8,9].

To date, few reports have quantitatively documented the immediate effects of orthosis use on dynamic balance in CC, and the impact of forced correction remains unclear. This case report aimed to quantitatively evaluate the immediate effects of orthosis-induced postural correction on static and dynamic balance in PD-associated CC using three-dimensional motion analysis. We also examined whether this intervention compromises compensatory stabilizing mechanisms.

## Case presentation

History and clinical findings

The patient was a man in his 80s with an eight-year history of PD and Hoehn & Yahr stage 3. The Movement Disorder Society-Unified Parkinson’s Disease Rating Scale (MDS-UPDRS) Part III score was 3 for both the posture and postural stability items. He presented with typical upper CC (thoracic fulcrum) accompanied by mild right lateral flexion. His daily medication regimen included levodopa/carbidopa, selegiline, zonisamide, and opicapone (levodopa equivalent daily dose: 950 mg). No apparent motor fluctuations were observed, and his physical function remained stable throughout the day.

Therapeutic intervention and assessment

**Figure 1:**
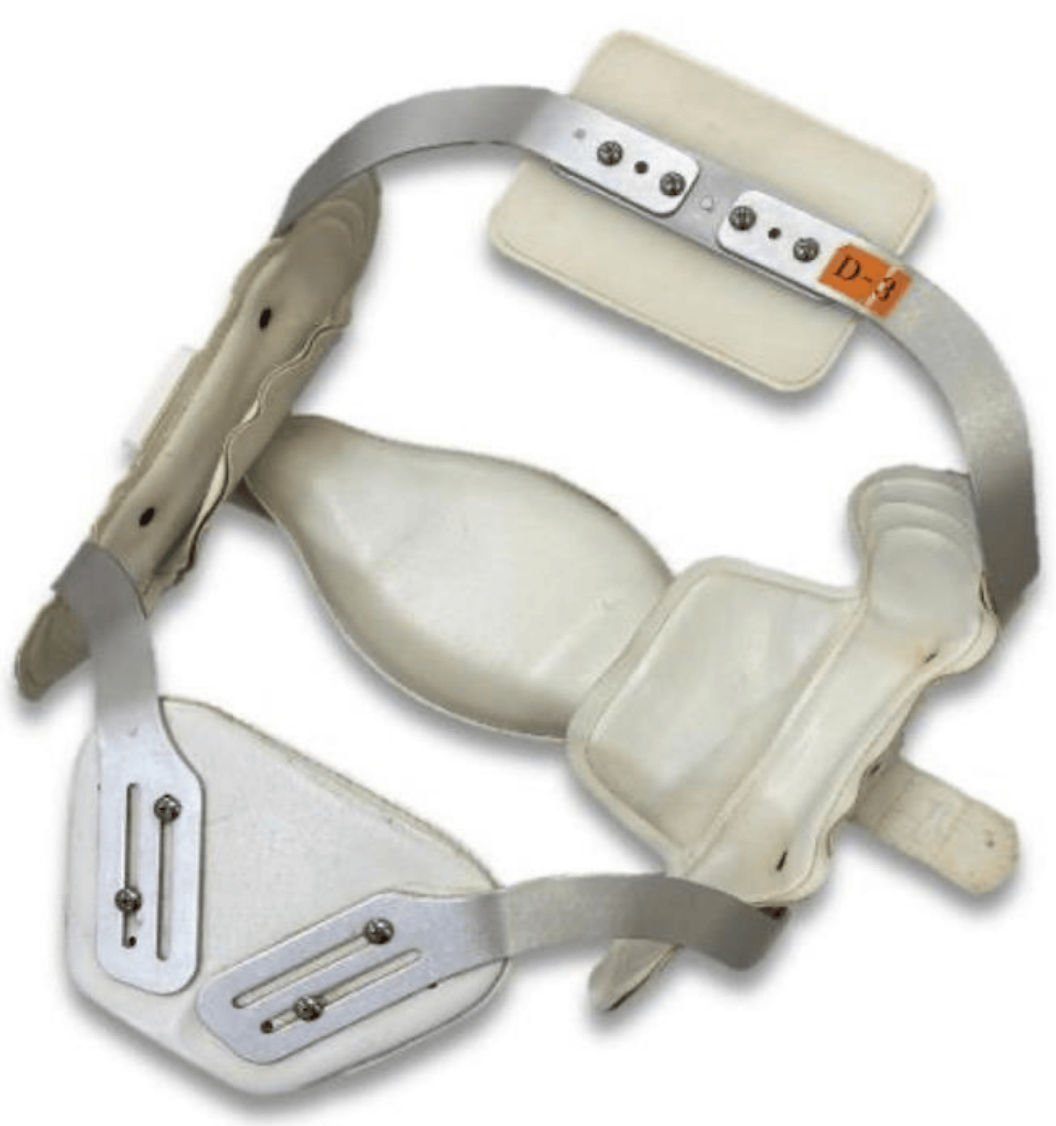
The Jewett brace used in the study. This figure shows the thoracolumbar orthosis (Jewett brace) used for the intervention. The device was managed by the Department of Rehabilitation and was fitted to the patient before assessment to ensure optimal alignment.

**Figure 2:**
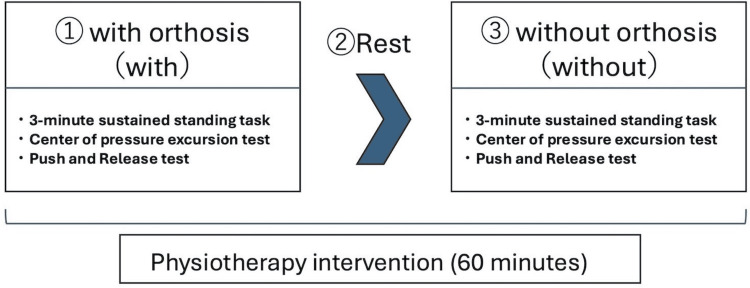
Flowchart of the experimental procedure. The assessment was conducted within a single 60-minute physiotherapy session. The patient first underwent the “with orthosis” condition (With), followed by a sufficient rest period to minimize fatigue. The same assessments were then repeated in the “without orthosis” condition (Without). The assessments included a three-minute sustained standing task, the Center of Gravity (COG) excursion test, and the Push and Release Test.

A Jewett brace was selected to promote thoracic extension (Figure 1). We assessed the immediate effects during a single 60-minute physiotherapy session. The experimental procedure is outlined in Figure 2. Assessments were conducted first in the “with orthosis” condition (With). After a sufficient rest period to minimize fatigue, the assessments were repeated in the “without orthosis” condition (Without).

Outcome measures

Balance and postural alignment were quantitatively assessed using a three-dimensional motion analysis system (Vicon, Oxford Metrics Ltd., Oxford, UK). The evaluation consisted of three tasks. First, a three-minute standing task was performed to measure the progression of CC; the change in trunk flexion angle was calculated from the start to the end of the three-minute period. Second, a Center of Gravity (COG) sway test was conducted to evaluate the limits of stability. The patient stood naturally and performed maximal voluntary weight shifts in the anterior, posterior, left, and right directions; the maximum displacement distance was calculated for each direction. Finally, the Push and Release Test (PRT) was performed five times per condition to assess reactive stepping responses [10]. Using motion-capture data, three temporal variables were analyzed: step initiation (time from release to the onset of the first step), first contact (time from release to landing of the first step), and final stabilization (time from release until cessation of the final compensatory step). Statistical analyses were performed using the 'scan' package in R software (version 4. 2. 2; R Foundation for Statistical Computing, Vienna, Austria) [11]. PRT time variables were compared between conditions using piecewise regression analysis, focusing on the immediate level effect to determine whether performance differed immediately following removal of the orthosis.

Outcomes

**Figure 3:**
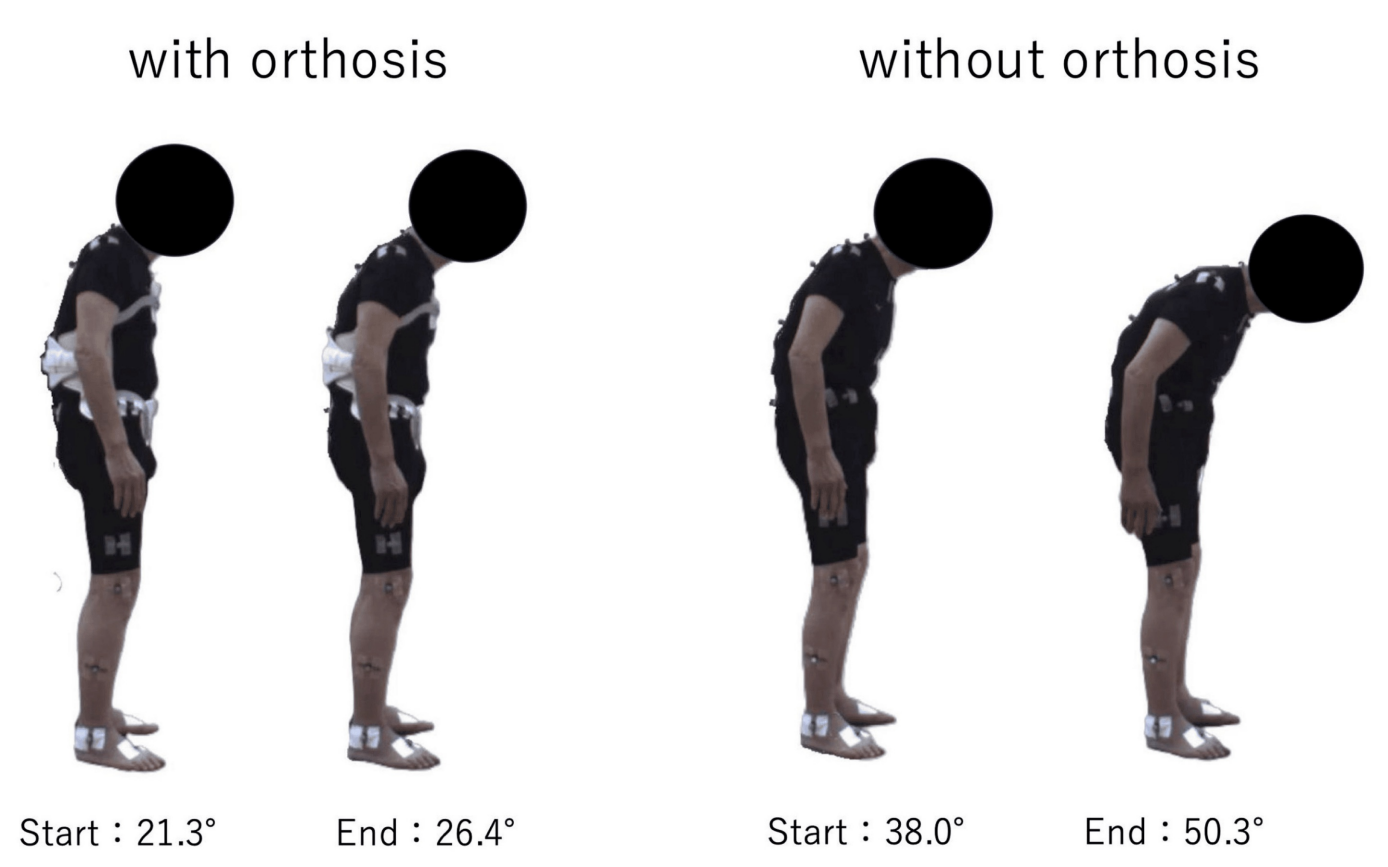
Changes in trunk flexion angle during the three-minute standing task. Visual comparison of posture at the start and end of the three-minute standing task. The left panel shows the “with orthosis” condition, in which the increase in trunk flexion was attenuated (start: 21.3°, end: 26.4°). The right panel shows the “without orthosis” condition, in which a larger increase in flexion was observed (start: 38.0°, end: 50.3°).

In the three-minute standing task in the “With” condition, the trunk flexion angle increased from 21.3° to 26.4°, with a change of +5.1°. In contrast, in the “Without” condition, it increased from 38.0° to 50.3°, with a change of +12.3°. With orthosis use, the change in trunk flexion angle decreased by 7.2° (Without-With), corresponding to a 58.5% attenuation compared with the Without condition (Figure 3 and Table 1). Notably, the change in trunk flexion angle in both conditions exceeded the minimal detectable change for the total CC angle reported by Schlenstedt et al. (3.7°); however, the angular change was larger in the “Without” condition [12].

**Table 1:** Comparison of postural alignment and dynamic balance outcomes with and without the orthosis. Push and Release Test values are presented as mean ± standard deviation, with the full range (minimum-maximum) shown in parentheses. The “Change” in trunk flexion angle represents the progression of forward flexion from the start to the end of the three-minute standing task. Percent attenuation of the increase in trunk flexion angle was calculated as (ChangeWithout-ChangeWith)/ChangeWithout × 100. Center of Gravity sway values indicate the maximal displacement distance achieved in each direction.

Outcome Measures	With Orthosis	Without Orthosis
Trunk Flexion Angle (degrees)		
Start	21.3	38
End	26.4	50.3
Change	+5.1 (58.5%)	12.3
Center of Gravity Sway (mm)		
Anterior	62	65
Posterior	63	65
Right	58	69
Left	31	40
Push and Release Test (seconds)		
Step initiation	0.18 ± 0.01 (0.17-0.19)	0.19 ± 0.05 (0.15-0.27)
First contact	0.46 ± 0.02 (0.44-0.49)	0.42 ± 0.06 (0.35-0.49)
Final stabilization	1.98 ± 0.67 (1.08-2.87)	2.51 ± 0.70 (1.70-3.31)

For the COG sway test, maximal displacement distances were comparable between conditions. In the “With” condition, displacement distances were 62 mm anteriorly, 63 mm posteriorly, 58 mm to the right, and 31 mm to the left. In the “Without” condition, displacement distances were 65 mm anteriorly, 65 mm posteriorly, 69 mm to the right, and 40 mm to the left (Table 1).

**Figure 4:**
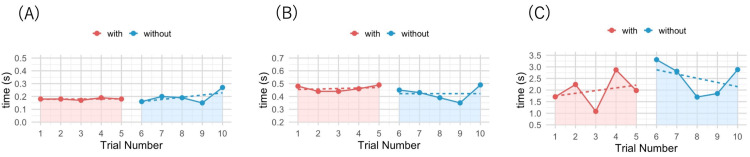
Results of the Push and Release Test (PRT). (A) Step initiation: time from release to the onset of the first step. (B) First contact: time from release to landing of the first step. (C) Final stabilization: time from release until cessation of the final compensatory step. The red markers indicate the “with orthosis” condition (trials 1-5), and the blue markers indicate the “without orthosis” condition (trials 6-10). The dashed lines represent regression lines used to evaluate trends within each condition.

For the PRT, step initiation time for the “With” condition was 0.18 ± 0.01 s (range: 0.17-0.19 s), and that for the “Without” condition was 0.19 ± 0.05 s (range: 0.15-0.27 s) (Figure 4A). Piecewise regression analysis revealed no significant difference between the two conditions (B = -0.02; 95% confidence interval (CI): -0.10 to 0.06; p = 0.60). Similarly, first contact time was 0.46 ± 0.02 s (range: 0.44-0.49 s) for the “With” condition and 0.42 ± 0.06 s (range: 0.35-0.49 s) for the “Without” condition (Figure 4B), whereas final stabilization time was 1.98 ± 0.67 s (range: 1.08-2.87 s) and 2.51 ± 0.70 s (range: 1.70-3.31 s), respectively (Figure 4C). Piecewise regression analysis showed no significant between-condition differences for either variable (first contact time: B = -0.05; 95% CI: -0.17 to 0.07; p = 0.43; final stabilization time: B = 0.55; 95% CI: -1.33 to 2.42; p = 0.59) (Table 1).

## Discussion

In this case, the Jewett brace attenuated the increase in trunk flexion during sustained standing, suggesting its involvement in maintaining a more upright posture in upper CC. One clinical concern when applying trunk orthoses for CC is the potential disruption of compensatory postural strategies. Previous computational modeling studies have hypothesized that abnormal postures in PD, including CC, might minimize postural sway in the presence of hypertonia [7]. If this compensatory mechanism was essential for our patient, mechanically correcting spinal alignment could have compromised dynamic balance or reactive stepping. However, we did not detect deterioration in COG sway limits or reactive stepping performance as measured by the PRT with the orthosis.

The relationship between postural correction and balance in CC warrants careful consideration from the aspect of existing literature. Whereas trunk orthoses are established as a non-pharmacological option for mechanical alignment [6], previous studies have largely focused on static outcomes or combined interventions. For instance, de Sèze et al. [6] reported that a program combining orthosis use with physiotherapy improved posture. However, the isolated, immediate effect of rigid bracing on dynamic stability has been reported less frequently. Our results are in contrast with the theoretical risks suggested by computational models [7]; in this case, rigid fixation did not appear to impede the compensatory stepping reactions required for fall prevention. This aligns with the perspective that the orthosis may have functioned similarly to intrinsic rigidity, often described as a compensatory strategy in PD.

The positive outcome may also reflect the biomechanical disadvantages imposed by the deformity itself. As suggested by Geroin et al. [8] and Urakami et al. [9], severe trunk flexion can be destabilizing owing to excessive lowering of the center of mass or limiting of hip extension. By improving alignment, the orthosis could mitigate these biomechanical constraints, thereby offsetting any loss of compensatory flexion. Consequently, in this case, the Jewett brace improved spinal alignment without negatively affecting dynamic balance.

Notably, step initiation times with orthosis were consistent across trials, exhibiting less variability than those without orthosis and thus demonstrating a stable, predictable response. This pattern suggests that the orthosis may have provided a consistent mechanical baseline, allowing the patient to generate protective steps more reliably. Importantly, the brace did not delay step initiation, addressing the clinical concern that trunk restriction might hinder the rapid stepping responses required for balance recovery. However, because testing was performed in a fixed order, practice or order effects might also have contributed to this pattern.

This study is limited by its single-case design and the small number of PRT trials. In addition, manual perturbations in the PRT have inherent variability, and assessments were performed in a fixed order (with orthosis followed by without orthosis). Although a resting period was included between the conditions, fatigue or order effects cannot be ruled out. In this case, we proceeded with the assessments and intervention after confirming that the patient did not report prominent diurnal motor fluctuations. However, in patients with PD, clear ON/OFF fluctuations related to levodopa wearing-off are common, and medication status may affect evaluation outcomes. Therefore, future studies should incorporate a standardized assessment timing while considering medication intake and using clinical symptom measures (e.g., UPDRS) in combination to validate the orthosis intervention outcomes. Trunk extension support using a Jewett brace represents a form of supportive management for CC and is not a direct treatment that modifies the underlying pathology. In addition, the availability of the orthosis and the resources for fabrication and fitting/adjustment might vary across institutions, imposing practical constraints on its clinical application. Future studies with larger sample sizes and randomized designs are needed to support the generalizability of the present findings.

## Conclusions

In this case, the Jewett brace attenuated the increase in trunk flexion during standing without compromising dynamic balance function in a patient with PD-associated CC. Although orthoses could theoretically hinder compensatory flexion, this case suggests that they may be applied safely. Our results indicate that the benefits of reducing the destabilizing effects of severe flexion may outweigh the risks of disrupting compensatory strategies. However, given the heterogeneity of PD, clinicians should carefully evaluate dynamic balance and stepping reactions on an individual basis when prescribing trunk orthoses.
